# Correlates of Maternal Iron Stores During Pregnancy and Cord Blood Ferritin Levels at Delivery in a Nigerian Tertiary Hospital

**DOI:** 10.7759/cureus.105023

**Published:** 2026-03-11

**Authors:** Chimaobi Nwankpa, John Imaralu, Adebayo Akadri, John Sotunsa, Elizabeth Grillo, Victor C Okebalama, Adesola Adekoya, Ngozi Adefala, Odutola Odugbemi, Blessing Nwankpa, Oluwuyiwa Adelowo, Nneoma Obinna-Chinatu, Rukayat Olayemi, Oluyemisi Okwudishu, Ekeleoma Obasi, Ugochukwu Chigozie

**Affiliations:** 1 Department of Obstetrics and Gynaecology, Babcock University Teaching Hospital, Ilishan-Remo, NGA; 2 Department of Histopathology, Babcock University Teaching Hospital, Ilishan-Remo, NGA; 3 Department of Paediatrics, Babcock University Teaching Hospital, Ilishan-Remo, NGA; 4 Department of Community Medicine, Babcock University Teaching Hospital, Ilishan-Remo, NGA; 5 Department of Obstetrics and Gynaecology, Manchester University NHS Foundation Trust, Manchester, GBR; 6 Department of Paediatrics, Royal Stoke University Hospital, University Hospitals of North Midlands NHS Trust, Stoke, GBR; 7 Department of Obstetrics and Gynaecology, Crescent University, Abeokuta, NGA; 8 Department of Internal Medicine, Babcock University Teaching Hospital, Ilishan-Remo, NGA; 9 Department of General Medicine, Royal Care Specialist Hospital, Abuja, NGA

**Keywords:** apgar score, birth weight, cord ferritin, delivery, iron stores, placenta weight, pregnancy, relationship

## Abstract

Background: Insufficient iron is a common nutritional deficiency globally. It is a significant cause of anemia in pregnancy and a common problem in obstetric care due to the increased demand for iron by the developing fetus and placenta. It has been associated with adverse maternal and perinatal outcomes. To maintain iron balance, maternal iron stores before and during pregnancy have been suggested to be an indispensable factor. However, there is no consensus on the effect of iron stores in mothers on neonatal iron stores.

Aim: To establish the relationship between maternal iron stores in pregnancy and cord ferritin at delivery, birth weight, placenta weight, and Apgar scores.

Methodology: This study included 170 mothers and neonatal pairs. Maternal venous blood was obtained in labor, while 5 ml of cord blood was collected soon after childbirth for ferritin and hemoglobin assay using enzyme-linked immunosorbent assay (ELISA) and a hemoglobinometer, respectively. Sociodemographic details, birth weight, placenta weight, and Apgar scores were obtained. IBM SPSS version 22 (IBM Corp., Armonk, NY) was employed to carry out the statistical data analysis.

Result: The prevalence of iron deficiency was 27.6% (47). The mean maternal ferritin concentration was 23.9 ± 19.8 ng/ml. The mean cord ferritin was 223 ± 234 ng/ml. There was no relationship between maternal ferritin levels and cord ferritin (r = 0.002, p = 0.981), cord hemoglobin, placenta weight, birth weight, and Apgar score. Cord ferritin and cord hemoglobin showed a negative correlation (r = -0.222, P = 0.004). The mean hemoglobin level (14.3 ± 1.9) was significantly higher in neonates with lower ferritin (t = 2.960, p = 0.004). Apgar scores at one and five minutes were significantly lower in neonates with low cord blood ferritin compared to neonates having normal blood ferritin (at one minute: 7 ± 1 vs. 8 ± 1, p = 0.039; at five minutes: 9 ± 1 vs. 10 ± 1, p = 0.011).

Conclusion: Maternal iron stores and neonatal iron stores showed no relationship between them at delivery. The inverse relationship between cord ferritin and neonatal hemoglobin suggests that some unexplained factors may be responsible for this, which warrants further research. Furthermore, adequate iron stores influenced better neonatal outcomes.

## Introduction

Iron deficiency is a global health problem affecting all regions, with developing countries being the most severely impacted. It is associated with adverse maternal and perinatal outcomes [1]. It has been reported that 41.8% of pregnant women are anemic globally; the African continent as a whole has a higher prevalence of 55.8%, of which iron deficiency is the major etiology in all. This poses a significant concern in low-income countries [2,3]. A prevalence ranging from 25% to 45.6% has been recorded in Nigeria [4,5].

Globally, iron deficiency is implicated as the etiology in more than half of cases of anemia in pregnancy [2]. In developing countries, nutritional deficiencies, malaria, and hookworm infestations are major causes of iron deficiency [2,6].

The World Health Organization (WHO) recommends that pregnant women be supplemented with iron during antenatal care for a positive pregnancy experience [7,8]. With worsening iron deficiency during pregnancy, women stand the risk of anemia with consequent poor pregnancy outcomes, such as increased susceptibility to infections, maternal anemia, low birth weight, preterm delivery, iron deficiency in infancy, and neurodevelopmental impairment in infants [9,10].

The most widely used biomarker for the assessment of iron stores is serum ferritin [2]. Although the assessment of bone marrow iron is the gold standard and an indicator that provides estimates of the size of stainable bone marrow iron, it is not a practical tool for measurement due to the invasiveness of the procedure [11]. Serum ferritin is a glycoprotein that binds to iron. It is a reflection of iron stores when features of inflammation are absent and may pose a diagnostic dilemma in such an instance. With the decline in iron stores, it is the first laboratory test to become abnormal and is not affected by recent ingestion of iron [12,13]. Hemoglobin estimation is routinely used in the assessment of the hematologic status of a woman while pregnant. It is used to make a diagnosis of anemia in pregnancy and not iron deficiency. Anemia in pregnancy occurs when hemoglobin concentration is <11 g/dL in the 1st and 3rd trimesters or <10.5 g/dL in the 2nd trimester. A threshold of 10 g/dL is acceptable as a cutoff value in developing countries; this is because studies have shown that adverse fetomaternal outcomes are not found when the hemoglobin concentration is above this level [2,14]. The use of hemoglobin can also indicate iron depletion, but it is a late marker. This is because in the early stage of iron deficiency, in which there is a loss of bone marrow iron stores, hemoglobin concentration and serum iron level remain normal, but ferritin falls. With the subsequent reduction in hemoglobin concentration, the level of iron falls further in the later stage. Hence, iron deficiency progressively results in iron deficiency anemia [13]. The widely accepted thresholds for iron deficiency using serum ferritin are <12 and <15 ng/mL in all stages of pregnancy [12,15,16].

Adediran et al. in Lagos, Nigeria, found that cord ferritin of anemic mothers was lower than that of non-anemic mothers, which revealed a significant association between maternal anemia and cord blood ferritin concentrations. This study was the only study that compared maternal iron stores and cord blood ferritin; however, there was no correlational analysis done to establish a relationship in this study. This was identified as a gap [17].

In India, a study conducted on 195 mother and neonate pairs by Swetha et al. (2017) showed a significant positive correlation between maternal and neonatal iron levels (r = 0.294, p = 0.000) [18].

Consequently, Terefe et al., studying the effect of maternal iron deficiency anemia on newborns' iron stores in Ethiopia, concluded that newborns born to mothers with iron deficiency anemia had significantly lower serum ferritin (P = 0.017) and hemoglobin concentrations (P = 0.024) [19]. A study done by Shukla et al. in India on 180 mother-infant pairs also showed a significant correlation between maternal and infant hemoglobin and ferritin levels at birth and 14 weeks after birth [20].

On the contrary, Emery and Barry in New Zealand revealed no statistical relationship between maternal and fetal cord blood for hemoglobin, iron, and ferritin levels [21]. Akhter et al.'s study on pregnant women and their neonate pairs found no significant correlation between maternal hemoglobin and cord hemoglobin quantities, but maternal serum ferritin demonstrated a positive relationship with cord ferritin (r = 0.94; P < 0.001) as well as with the weight of the placenta [22].

Consequently, there is conflicting evidence regarding the relationship between maternal iron stores and neonatal iron status at delivery. Therefore, this study's objectives were to determine the prevalence of iron deficiency in Babcock University Teaching Hospital, Ilishan-Remo, Ogun State, Nigeria, and to determine the relationship between maternal iron stores in pregnancy and cord blood serum ferritin at delivery.

## Materials and methods

This study was a cross-sectional scientific research carried out in Babcock University Teaching Hospital (BUTH), a private Nigerian tertiary health institution located in Ilishan-Remo, Ogun State. The hospital is a faith-based tertiary health institution that provides care to patients in Ogun State, its suburban areas, and neighboring states. Ilishan-Remo is a semi-urban locality situated in the Ogun East senatorial district within Ogun State, Nigeria. The target population was pregnant women from Ilishan-Remo who presented for delivery at BUTH with their neonates and met the inclusion criteria. The exclusion criteria included unbooked patients, women with multiple pregnancies, hemoglobinopathies, chronic diseases, sepsis, liver disease, antepartum hemorrhage, bleeding four weeks prior, prolonged labor, obstructed labor, and birth asphyxia. All women were supplemented in the antenatal clinic with Ferofer® oral iron daily, containing 120 mg elemental iron, 1.5 mg folic acid, 61.8 mg zinc sulfate, 15 mcg cyanocobalamin, and 100 mg vitamin C.

Sample size determination

The Leslie Kish formula ($N = Z^{2} p(1-p) / d^{2}$) was applied to ascertain the minimal size of the sample needed for the research [23]. In a study of cord ferritin and hemoglobin conducted in Lagos, Nigeria, the prevalence of iron deficiency was 11.2% [13]. The estimated sample size for the research was 170.

Inclusion and exclusion criteria

The inclusion criteria of the study were all eligible women at delivery and term who gave consent to be part of the study and those with live-birth neonates at delivery, while the exclusion criteria were multiple pregnancies, unbooked patients, hemoglobinopathies, women with chronic diseases (e.g., retroviral disease, tuberculosis, diabetes mellitus, and chronic kidney disease), pregnant women with fever, pregnant women with liver disease, women with antepartum hemorrhage, and women who had been transfused within the last four weeks before delivery.

Data collection and laboratory procedures

The study started in July 2021 and ended in January 2022. The antenatal case notes of consenting, booked pregnant women in labor were reviewed for eligibility. Socio-demographic details of the women and obstetric parameters were obtained. Venous blood (5 ml) was collected from the antecubital vein of the preferred maternal arm within one hour of presentation for ferritin and hemoglobin estimation. At the delivery of the baby, the umbilical cord was double clamped and cut between the two clamps. The clamp on the umbilical cord linking to the placenta, which was still attached to the uterus, was gently released to passively allow cord blood to flow into a sample bottle held by an assistant. This was done after administration of oxytocin but before delivery of the placenta. Blood sample (2 ml) was dispensed into a disodium ethylene diamine tetra-acetic acid (EDTA) bottle for hemoglobin estimation, and 3 ml of blood was dispensed into a plain vacutainer bottle for serum ferritin assay for each of the samples, respectively. After centrifugation at 5000 rpm, the serum samples were stored at -200°C and analyzed within five months using a ferritin enzyme-linked immunosorbent assay (ELISA) kit from Monocent® (Northridge, CA).

Serum ferritin principle

The solid-phase immunosorbent assay, which uses an enzyme, serves as the foundation for the quantitative ferritin analysis kit. The assay method uses an antibody in the antibody-enzyme conjugate solution, along with an additional mouse monoclonal anti-ferritin antibody for solid-phase immobilization. When the examination material and the antibodies were allowed to undergo reactions in tandem, ferritin molecules were positioned at the interphase of the solid phase and the enzyme-linked antibodies. When the tetramethylbenzidine (TMB) solution was added and incubated, a purple color developed, the magnitude of which corresponded precisely with the serum ferritin level. A spectrophotometer was used to measure this at a wavelength of 450 nm.

The normative value for maternal ferritin in pregnancy is 12-150 ng/mL. It has been reported that the adverse effects of iron deficiency begin to present below a cut-off of 12 ng/mL. There is no consensus on the cutoff for cord ferritin. It has been documented that cord ferritin increases with advancing gestational age. The cutoff value used in this study is 160 ng/mL, based on mean ferritin levels obtained among term neonates [24,25].

Data analysis

IBM SPSS version 22 (IBM Corp., Armonk, NY) was used to analyze the data, which were then displayed in graphical forms as frequencies and percentages. Continuous parameters were compared using the Student's t-test, while categorical parameters were compared using the chi-square test. Maternal ferritin, cord blood ferritin, and fetal outcome indicators such as weight at delivery, weight of placenta, and Apgar score were all correlated using Pearson's product-moment correlation coefficient. The significance level, or P-value, was established at P < 0.05.

Ethical consideration

The BUTH’s Research Ethics Panel granted ethical authorization for this project (BUHREC 368/20). Participants received an in-depth description of the research project, and informed consent was acquired prior to enrolment in the scientific investigation, while upholding absolute privacy of the data collected.

## Results

Table 1 highlights the sociodemographic characteristics of the mothers who participated in this study. A total of 53 (31.2%) were aged 25-29 years. The majority had at least one parous experience, with more than half (93, 54.7%) of the women within parity of 1-4. Primigravidae were 74 (43.5%), while only three (1.8%) were grand-multiparous. Most (168, 98.8%) of the study participants were married. Of all the respondents, 111 (65.3%) had a tertiary education, while two (1.2%) had no formal education.

**Table 1:** Sociodemographic characteristics of the study participants.

Sociodemographic characteristics	Frequency (N = 170)	Percentage (%)
Age groups (years)		
18-24	33	19.4
25-29	53	31.2
30-34	48	28.2
≥35	36	21.2
Parity		
0	74	43.5
1-4	93	54.7
≥5	3	1.8
Religion		
Christianity	146	85.9
Islam	24	14.1
Traditional	-	-
Educational status		
None	1.2	1.2
Primary	2.9	2.9
Secondary	30.6	30.6
Tertiary	65.3	65.3
Ethnicity		
Yoruba	120	70.6
Igbo	36	21.2
Hausa	4	2.4
Others	10	5.9
Marital status		
Single	2	1.2
Married	168	98.8
Separated	-	-
Client's occupation		
Unskilled	24	14.1
Semiskilled	83	48.8
Skilled	63	37.1
Husband's occupation		
Unskilled	16	9.4
Semiskilled	69	40.6
Skilled	85	50.0

**Figure 1:**
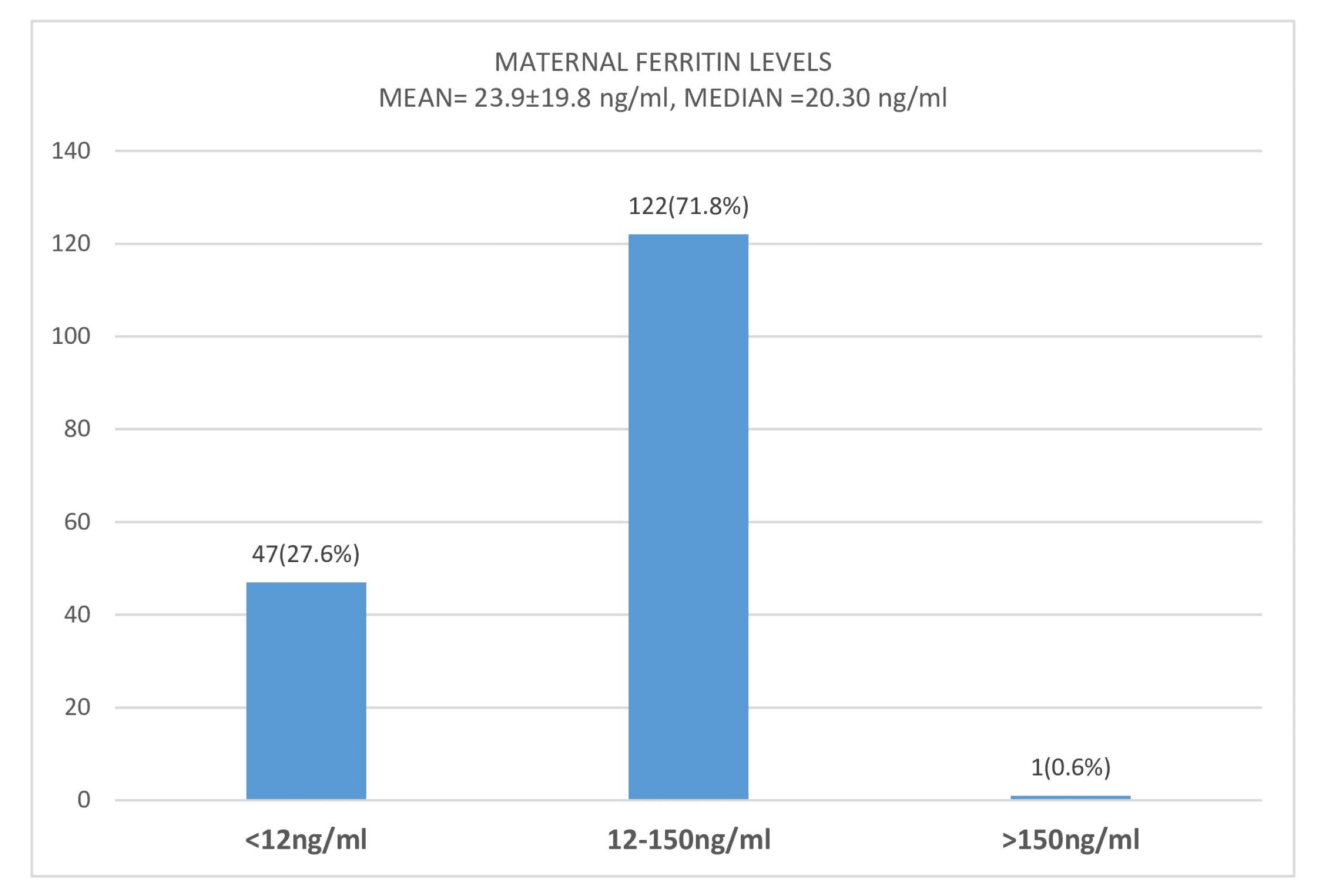
Bar chart showing categorized levels of maternal ferritin among 170 study participants.

Figure 1 shows the categorized levels of maternal ferritin of the study participants. The mean maternal ferritin level was 23.9 ± 19.8 ng/ml, whereas the median ferritin level was 20.30 ng/ml. The prevalence of iron deficiency was 27.6%, estimated using a cutoff of 12 ng/mL [26].

Table 2 highlights the relationship between the sociodemographic characteristics of the study participants and their serum ferritin levels. The sociodemographic characteristics and maternal serum ferritin levels did not correlate statistically significantly. Although not statistically significant, there was a higher prevalence of low maternal ferritin levels among those in unskilled labor and with lower levels of education.

**Table 2:** Relationship between sociodemographic variables and maternal serum ferritin level using the chi-square test.

Sociodemographic characteristics	Hypoferritinemia (Total = 47)	Normal ferritin (Total = 123)	χ^2^	P-value
	N (%)	N (%)		
Age groups (years)				
18-24	11 (33.3)	22 (66.7)		
25-29	16 (30.2)	37 (69.8)	1.923	0.589
30-34	13 (27.1)	35 (72.9)		
≥35	07 (19.4)	29 (80.6)		
Parity				
0	21 (28.4)	53 (71.6)		
1-4	25 (26.9)	68 (73.1)	0.096	0.953
≥5	1 (33.3)	2 (66.7)		
Religion				
Christianity	40 (27.4)	106 (72.6)	0.032	0.811
Islam	07 (29.2)	17 (70.8)		
Traditional	-	-		
Educational status				
None	1 (50.0)	1 (50.0)		
Primary	2 (40.0)	3 (60.0)	1.460	0.692
Secondary	16 (30.8)	36 (69.2)		
Tertiary	28 (25.2)	83 (74.8)		
Ethnicity				
Yoruba	32 (26.7)	88 (73.3)		
Igbo	13 (36.1)	23 (63.9)	3.168	0.366
Hausa	-	4 (100.0)		
Others	2 (20.0)	8 (80.0)		
Marital status				
Single	1 (50.0)	1 (50.0)	0.506	0.478
Married	46 (27.4)	122 (72.6)		
Separated	-	-		
Client's occupation				
Unskilled	10 (41.7)	14 (58.3)		
Semiskilled	21 (25.3)	62 (74.7)	2.746	0.255
Skilled	16 (25.4)	47 (74.6)		
Husband’s occupation				
Unskilled	5 (31.3)	11 (68.8)		
Semiskilled	23 (27.1)	62 (72.9)	0.119	0.942
Skilled	19 (27.5)	50 (72.5)		

**Figure 2:**
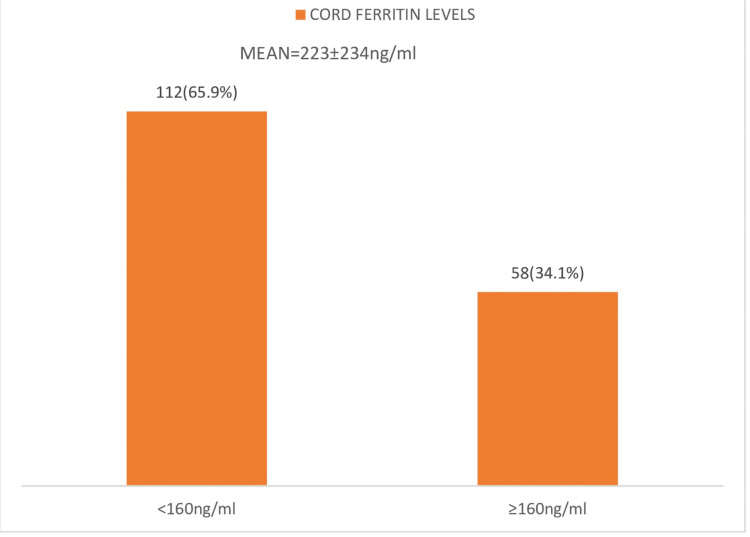
Bar chart showing categorized levels of cord blood ferritin among 170 study participants.

Figure 2 shows that the majority (112, 65.9%) of neonates had low cord blood ferritin, measured using a cutoff of ≥160 ng/ml [24,25]. The mean ferritin was 223 ± 234 ng/ml.

Table 3 presents the Pearson correlation analysis of the relationship between maternal serum ferritin and neonatal outcomes. The table showed no statistically significant correlation between mother's serum ferritin level and newborn's outcomes, including cord ferritin (r = -0.002, P = 0.981), cord blood hemoglobin (r = 0.076, P = 0.323), weight of the placenta (r = 0.082, P = 0.287), weight at birth (r = 0.008, P = 0.914), and Apgar score.

**Table 3:** Correlation between maternal serum ferritin levels and neonatal outcomes using Pearson’s correlation coefficient.

Variables	R-value	P-value
Cord blood serum ferritin (ng/ml)	-0.002	0.981
Cord blood hemoglobin (g/dl)	0.076	0.323
Placenta weight (kg)	0.082	0.287
Birth weight (kg)	0.008	0.914
Apgar score at 1 minute	0.040	0.601
Apgar score at 5 minutes	-0.025	0.743

Table 4 shows a comparison between the level of cord blood ferritin (low/normal) and neonatal outcome measures. The mean hemoglobin concentration in those with low cord blood ferritin was significantly higher than that of those with normal ferritin (14.3 ± 1.9 vs. 13.4 ± 1.9, p = 0.004). However, at one and five minutes, Apgar scores were significantly lower in neonates with low cord blood ferritin (at one minute: 7 ± 1 vs. 8 ± 1, p = 0.039; at five minutes: 9 ± 1 vs. 10 ± 1, p = 0.011).

**Table 4:** Comparison between cord blood ferritin levels and neonatal outcome variables using the Student's t-test. * Statistically significant at p < 0.05.

Variables	Low ferritin (Mean ± SD) (N = 112)	Normal ferritin (Mean ± SD) (N = 58)	t-value	P-value
Hemoglobin (g/dL)	14.3 ± 1.9	13.4 ± 1.9	2.960	0.004*
Birth weight (kg)	3.2 ± 0.5	3.2 ± 0.4	0.025	0.980
Placental weight (kg)	0.7 ± 0.1	0.7 ± 0.1	0.000	1.000
Apgar at 1 minute	7 ± 1	8.0 ± 1	2.085	0.039*
Apgar at 5 minutes	9 ± 1	10 ± 1	2.570	0.011*

Scatter plots (not shown) revealed that cord blood ferritin and hemoglobin had a negative association (r = -0.222, p = 0.004). It also showed that cord blood ferritin and Apgar scores at five minutes had a positive association (r = 0.162, p = 0.035).

## Discussion

The mean maternal ferritin level in this study was within the normal range for ferritin levels in pregnancy. This was slightly higher than the findings reported in Abia but was lower than those reported in Zaria [27,28]. The result revealed the variations in ferritin levels among different ethnic groups with diverse diets, and thus indicates the need for monitoring and iron supplementation in pregnancy. This study also revealed the prevalence of hypoferritinemia to be 27.6% among the participants. This finding was comparable to the prevalence of 30% found in Cross River [29]. However, a study done in Lagos, which is an urban setting, observed a lower prevalence of 12.3% [2]. On the other hand, observations from rural environments in northeastern Nigeria showed a higher prevalence of hypoferritinemia among pregnant women, with the study reporting a prevalence as high as 90.0% [30].

Furthermore, a similar study conducted by Okafor et al. to evaluate the iron status of pregnant women in rural and urban communities of Cross River State, the southern part of Nigeria, observed that the incidence and severity of anemia and anemia caused by iron deficiency were found to be significantly greater among pregnant women from the countryside than those from cities [29]. This can be attributed to their level of education, as well as the precarious nutritional status of the pregnant women, noted from the fact that most of them were housewives with little or no official income [29]. This finding corroborates the observation in our study, where an increasing trend of hypoferritinemia was observed as the level of education decreased. In addition, it was also noted that the more unskilled the participants were, the higher the rate of hypoferritinemia. The location of this present study can be classified as semi-urban. Overall, it can be inferred that the settlement of the participants may have an influence on the magnitude of the problem. This may be due to factors such as poor access to quality healthcare, educational level, and affordability of adequate nutrition and supplements during pregnancy. It can thus be inferred that iron deficiency still prevails among our women, and more attention should be paid to supplementation.

Ferritin levels in cord blood and maternal ferritin at delivery did not correlate statistically significantly. Similar findings were observed in New Zealand, Bangladesh, and Brazil [21,22,31]. However, this was at variance with the findings in India, Ethiopia, and China [18,19,26].

There was a significant inverse relationship between cord blood ferritin and cord hemoglobin in this study. It was similar to the observation by Delaney et al. [32]. They hypothesized that the association between low cord hemoglobin and high cord blood ferritin levels suggests that certain newborns may be unable to utilize the iron stored in their serum ferritin for the synthesis of red blood cells [32].

Additionally, it has been demonstrated that erythrocytes in newborns exhibit metabolic traits, morphologies, and membrane compositions that are markedly different from those of erythrocytes in adults. These differences may change the capacity to use the stored iron for the formation of hemoglobin in utero [33]. Keeping a pregnant woman's nutritional condition at its best at all times is crucial since a variety of factors can influence how stored iron is used for hemoglobin production. The inverse relationship between hemoglobin and serum ferritin, however, is supported by some studies that use information related to these biomarkers in cord blood. These research investigations attribute this observation to a favored use of iron for erythropoiesis needs, particularly in cases where ferritin concentrations are decreased, and hemoglobin levels are comparatively greater [34]. Iron utilization is prioritized for erythropoietic needs at the cost of tissue and storage iron in the setting of a fetal hypoxic condition, according to neonatal autopsy investigations, which indicate that every 1 ng/mL of serum ferritin corresponds to 2.7 mg/kg of storage iron [35]. This emphasizes the urgency for pregnant women to take iron supplements.

The results of this study also showed a strong positive correlation between the Apgar scores of neonates and cord blood ferritin. This could suggest that an infant's health, particularly at birth, may be influenced by iron levels. Iron deficiency anemia can result from a lack of iron that lasts as long as 12 months, as shown by the neonate's diminished iron storage [36]. Given the potential long-term negative implications, this illness should be recognized and treated as soon as possible. For newborns at risk, vigilant observation and sufficient supplements are consequently necessary.

The study has multiple strengths, including its focus on an important maternal-fetal health issue, the direct measurement of both maternal iron status and cord blood ferritin levels at delivery, and the use of clinical data from a tertiary hospital, which provides practical relevance, among others. To reduce confounders and improve reliability of study outcomes, pregnant women with multiple pregnancies, unbooked patients, women with hemoglobinopathies and chronic diseases (e.g., retroviral disease, tuberculosis, diabetes mellitus, and chronic kidney disease), pregnant women with fever, liver disease, or antepartum hemorrhage, and those who had been transfused within the last four weeks prior to delivery were excluded from the study.

Regarding the limitations of this study, it was a single-center study and may not be a true representation of the general study population, and the study could not assess maternal ferritin levels pre-conception and at various trimesters. Potential confounding factors such as maternal nutrition, iron supplementation, infections, and gestational age, as well as possible limitations related to sample size and the study's observational design, are also limitations of this study.

## Conclusions

The prevalence of iron deficiency in this study was high. There was no statistically significant relationship between maternal iron stores and cord serum ferritin at delivery. Nevertheless, it showed that the cord blood had an elevated level of hypoferritinemia, which did not match the maternal serum levels at birth. The level of newborn hemoglobin and cord blood ferritin had a statistically significant negative association. The level of ferritin also significantly influenced the Apgar score of the infants, especially at five minutes. This shows that iron remains a nutrient of interest and must be optimized before and during pregnancy so as to optimize maternal and neonatal stores. It should be monitored in the mother and the infant because the work on neonatal iron stores and prevention of complications may actually begin early in pregnancy.

Therefore, we advise that public health initiatives to inform the public about the importance of a nutritious diet and iron supplementation prior to conception, or not later than the start of pregnancy, are becoming more and more necessary. Additionally, given the immediate and long-term implications, a routine investigation into the newborn ferritin level at birth should be encouraged.
